# DT‐678 inhibits platelet activation with lower tendency for bleeding compared to existing P2Y_12_ antagonists

**DOI:** 10.1002/prp2.509

**Published:** 2019-07-25

**Authors:** Dale A. Lauver, Dawn S. Kuszynski, Barbara D. Christian, Matthew P. Bernard, James P. Teuber, Bruce E. Markham, Yuqing E. Chen, Haoming Zhang

**Affiliations:** ^1^ Department of Pharmacology and Toxicology Michigan State University East Lansing MI USA; ^2^ Diapin Therapeutics, LLC Ann Arbor MI USA; ^3^ Department of Pharmacology University of Michigan Ann Arbor MI USA

**Keywords:** animals, bleeding time, clopidogrel, platelets, purinergic P2Y receptor antagonists, thrombosis

## Abstract

The novel clopidogrel conjugate, DT‐678, is an effective inhibitor of platelets and thrombosis in preclinical studies. However, a comparison of the bleeding risk with DT‐678 and currently approved P2Y_12_ antagonists has yet to be determined. The objective of this study was to evaluate the bleeding tendency of animals treated with clopidogrel, ticagrelor, and DT‐678. Ninety‐one New Zealand white rabbits were randomized to one of 13 treatment groups (n = 7). Platelet activation was assessed by flow cytometry and light transmission aggregometry before and after the administration of various doses of DT‐678, clopidogrel, and ticagrelor. Tongue template bleeding times were also measured before and after drug treatment. Treatment with P2Y_12_ receptor antagonists caused a dose‐dependent reduction in markers of platelet activation (P‐selectin and integrin α_IIb_β_3_) and aggregation in response to adenosine diphosphate stimulation. At the same doses required for platelet inhibition, clopidogrel and ticagrelor significantly prolonged bleeding times, while DT‐678 did not. DT‐678 and the FDA‐approved P2Y_12_ antagonists clopidogrel and ticagrelor are effective inhibitors of platelet activation and aggregation. However, unlike clopidogrel and ticagrelor, DT‐678 did not prolong bleeding times at equally effective antiplatelet doses. The results suggest a more favorable benefit/risk ratio for DT‐678 and potential utility as part of a dual antiplatelet therapy regimen.

**Abbreviations**
AA: arachidonic acid
ACS: acute coronary syndromes
ADP: adenosine diphosphate
AF: Alexa Fluor
AM: active metabolite
CYP450: cytochrome P450
DAPT: dual antiplatelet therapy
DMA: N,N‐dimethylacetamide
DPBS: Dulbecco's phosphate‐buffered saline
IM: intramuscular
PLATO: Platelet Inhibition and Patient Outcomes
PPP: platelet‐poor plasma
PRP: platelet‐rich plasma

## 1 INTRODUCTION

Together with aspirin, purinergic P2Y_12_ receptor antagonists, like clopidogrel and ticagrelor, are widely used in dual antiplatelet therapy (DAPT) for the prevention of thrombosis in patients with acute coronary syndrome (ACS).^1^, ^2^, ^3^, ^4^, ^5^ Approximately, one million patients receive DAPT for ACS in the United States every year. Recent clinical trials have demonstrated the benefits of DAPT beyond 1 year and it is anticipated that long‐term use of DAPT will steadily increase.^6^, ^7^ The most concerning adverse event associated with any antithrombotic therapy is bleeding. Head‐to‐head comparison of bleeding tendency between P2Y_12_ antagonists is difficult since the classification of severity and clinical relevance of bleeding events differ in many of the large clinical trials. Additionally, even minor bleeding events, while not life‐threatening in and of themselves, are significant since they are one of the most important reasons for antiplatelet therapy nonadherence which can leave patients at increased risk for thrombotic events.^8^ Despite the approval of newer, more efficacious agents, clopidogrel continues to be broadly used in clinical cardiology. The comparative bleeding safety of clopidogrel compared to the newer agents like prasugrel and ticagrelor has been demonstrated in multiple large‐scale clinical trials.^9^, ^10^ In the Platelet Inhibition and Patient Outcomes (PLATO) trial, ticagrelor significantly increased spontaneous bleeds, major bleeds, major plus minor bleeds, and major plus minor plus minimal bleeds compared to clopidogrel. Therefore, clopidogrel is the preferred agent for long‐term management of patients. Clopidogrel, however, is subject to several limitations which include interpatient variability, delayed onset of action, and frequent drug‐drug interactions.^11^, ^12^ In addition, approximately 30% of Caucasians and 60%‐70% of Asians fail to respond to clopidogrel therapy due to polymorphisms in CYP2C19.^11^, ^13^, ^14^ As a result, these patients have increased risk of major adverse cardiovascular events.^14^, ^15^

**Figure 1:**
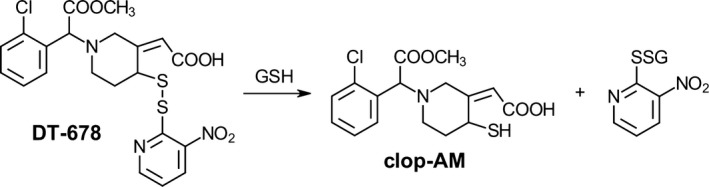
In the presence of glutathione (GSH), DT‐678 releases the clopidogrel active metabolite (clop‐AM) via a thiol exchange reaction without the requirement of CYP2C19

Our group has previously reported the development of DT‐678 (née ClopNPT), a disulfide conjugate of the clopidogrel active metabolite (AM) with 3‐nitropyridine‐2‐thiol.^16^, ^17^, ^18^ In the presence of glutathione, DT‐678 is readily converted to the AM through a disulfide exchange reaction as illustrated in Figure 1.^17^ Our earlier studies have demonstrated significant inhibition of ex vivo platelet aggregation and thrombosis in mice and rabbits.^16^, ^18^ Furthermore, we have established that DT‐678 releases the AM with a T_max_ of <5 minutes in C57BL/6 mice via both oral and intravenous routes, and the plasma concentrations of the AM reached C_max_ values of >1000 ng/mL after a 5 mg/kg intravenous dose or a 10 mg/kg oral dose.^18^ These results suggest that DT‐678 has favorable pharmacokinetic/pharmacodynamic properties that may potentially overcome the attenuated pharmacokinetic properties of clopidogrel and thus significantly improve the efficacy of antiplatelet therapy.

In this study, we sought to further evaluate the compound by comparing antiplatelet activity and bleeding tendency in animals treated with the approved P2Y_12_ antagonists clopidogrel and ticagrelor, or DT‐678. Our results demonstrate dose‐dependent inhibition of platelet aggregation and activation by all agents. However, bleeding times were significantly prolonged by clopidogrel and ticagrelor, but not DT‐678. These findings suggest that DT‐678 may be safer in the clinical setting while maintaining similar antiplatelet efficacy.

## 2 MATERIALS AND METHODS

### 2.1 Chemicals

Racemic DT‐678 was synthesized and purified to 99% by Beijing SL Pharmaceuticals. S‐clopidogrel and ticagrelor were purchased from Cayman Chemical Co. Alexa Fluor (AF) 647‐tagged anti‐CD62P antibody was purchased from MBL (D280‐A64) and fibrinogen‐FITC (fluorescein isothiocyanate) was purchased from Abcam (ab92788). Adenosine diphosphate (ADP), arachidonic acid (AA), and collagen were purchased from Chrono‐log Corporation. Polyethylene glycol 400 and N,N‐dimethylacetamide (DMA) were purchased from Millipore Sigma.

### 2.2 Animal care and use

The procedures used in this study were in accordance with the Michigan State University guidelines and were approved by the Institutional Animal Care and Use Committee (Animal Use Form 07/17‐115). Michigan State University Campus Animal Resources provided all veterinary care.

### 2.3 Surgical preparation of rabbits and administration of drugs

Ninety‐one male New Zealand white rabbits (1.9‐2.4 kg) were obtained from Charles River Laboratories, Inc (Wilmington, MA). All animals were acclimated for a minimum of 5 days and had free access to standard chow and fresh water prior to the study. Animals were maintained on an automated 12/12‐hour light/dark cycle with 7:00 am as the start of the light phase. On the day of the study, rabbits were sedated and anesthetized to surgical unconsciousness with ketamine (40 mg/kg, intramuscular [IM]), xylazine (5 mg/kg, IM), and isoflurane (1%‐3%, inhaled). The surgical site was shaved, and the rabbits were placed on a 37°C heating pad. Isoflurane was administered through a mask that was placed over the mouth and nose. The right jugular vein was surgically isolated and instrumented with a polyethylene cannula for drug administration and blood collection. Respiratory rate, the lead II electrocardiogram, heart rate, and body temperature were monitored throughout the procedure. Vehicle, DT‐678 (0.1‐3.0 mg/kg), clopidogrel (0.3‐10.0 mg/kg), or ticagrelor (0.1‐3.0 mg/kg) were administered via the jugular cannula (n = 7 per dose group). P2Y_12_ antagonists were dissolved in a 20:80 (v/v) mixture DMA and polyethylene glycol 400. Drugs were administered as an intravenous bolus injection at the indicated doses.

### 2.4 Collection of whole blood

Blood samples were collected from the jugular cannula into a syringe containing 3.2% sodium citrate as an anticoagulant (1:10 citrate to blood ratio) before (baseline) and 10 minutes, 1 hour, and 2 hours after drug treatment. The blood samples were divided into two parts: 1.5 mL was used to perform flow cytometry (baseline and 1 hour posttreatment time points only) while the remainder was used for platelet aggregometry (see below).

### 2.5 Determination of platelet activation by flow cytometry

Platelet activation was determined by anti‐CD62P‐AF647 and fibrinogen‐FITC binding in whole blood stimulated by ADP. Citrated blood (450 µL) was incubated with ADP (20 µmol/L) or HEPES‐buffered saline for 2 minutes. Fibrinogen‐FITC (0.17 mg/mL) was then added to these samples and incubated for 15 minutes in the dark. The blood was fixed with 1 mL of 1% paraformaldehyde for 15 minutes and washed with 1‐mL Dulbecco's phosphate‐buffered saline (DPBS). Subsequently, anti‐CD62P antibody (0.5 µg/mL) was added to the samples and incubated for 15 minutes followed by washing and resuspension in DPBS. Flow cytometric assessment was performed using a BD Accuri C6 (BD Biosciences) available in the MSU South Campus Flow Cytometry Core Facility. Events (20 000) were collected on log scale for FSC‐A and SSC‐A, gated on the platelet scatter‐based population, followed by doublet discrimination. Quadrant gates for fibrinogen‐FITC and anti‐CD62P‐AF647 positive events were generated based on fluorescence minus one control prepared for each animal and time point. Double‐positive (CD62P^+^fibrinogen^+^) platelets were quantified as a measure of platelet activation. Data were analyzed using CFlow Plus software, v1.0.227.04 (BD Biosciences).

### 2.6 Determination of platelet aggregation by light transmission aggregometry

Platelet reactivity was determined in platelet‐rich plasma (PRP) obtained from whole blood samples using light transmission aggregometry. Whole blood samples (see above) were centrifuged at 150 *g* for 10 minutes at room temperature and the supernatant was collected. The pellet was then centrifuged at 1500 *g* at room temperature for 10 minutes to obtain the platelet‐poor plasma (PPP). Ex vivo platelet aggregation was assessed using a 4 channel aggregometer (Chrono‐log Corporation Model 700; Chrono‐log Corporation). PRP was continually stirred and maintained at 37°C during the assay. The change in light transmission relative to PPP after stimulation with platelet agonists (ADP [20 µmol/L], AA [500 µmol/L], and collagen [2 µg/mL]) was recorded.

### 2.7 Determination of bleeding time in New Zealand white rabbits

To evaluate the bleeding risk of the P2Y_12_ antagonists, bleeding times were measured using a Surgicutt^®^ device (Accriva Diagnostics), which creates a uniform 5‐mm long and 1‐mm deep incision on the upper surface of the tongue. The margins of the lesion were blotted every 10 seconds with filter paper until blood was no longer transferred from the tongue to the filter paper. The interval from the time the incision was created to the time that blood was no longer apparent on the filter paper is considered the tongue bleeding time. Bleeding times were assessed before treatment and 2 hours after treatment.

### 2.8 Statistical analysis

Data were analyzed using GraphPad Prism 7 software (GraphPad Software) and are presented as mean ± SEM. Statistical differences between drug treatment groups and vehicle were analyzed by one‐way ANOVA followed by Dunnett's multiple comparison test. Results were considered significant at *P < .05, **P < .01, ***P < .001, and ****P < .0001.

## 3 RESULTS

### 3.1 P2Y_12_ antagonists decrease α‐granule secretion and the formation of integrin α_IIb_β_3_

**Figure 2:**
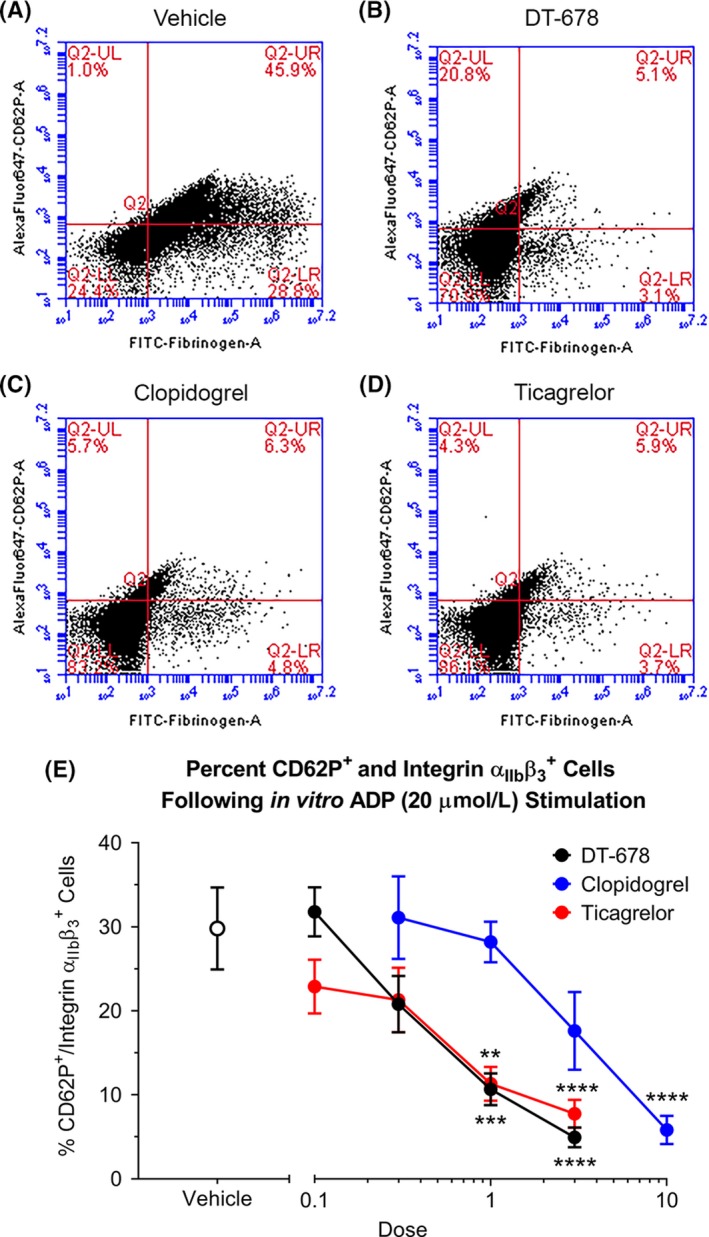
Flow cytometric assessment of platelets activated with ADP. Representative scatter plots of platelets from animals treated with (A) vehicle, (B) 3.0 mg/kg DT‐678, (C) 10.0 mg/kg clopidogrel, and (D) 3.0 mg/kg ticagrelor. (E) Pretreatment with antiplatelet agents caused a dose‐dependent reduction in α‐granule secretion (as measured by CD62P expression) and the formation of integrin α_IIb_β_3_ (indicated by FITC‐fibrinogen binding) in response to ADP activation. Double‐positive (CD62P^+^fibrinogen^+^) events were quantified in the upper right quadrant of individual animal flow cytometric dots plots. The data are presented as the mean ± SEM of seven separate experiments. **P < .01, ***P < .001, ****P < .0001 when compared with vehicle‐treated group by one‐way ANOVA followed by Dunnett's post hoc test. ADP, adenosine diphosphate

The effects of P2Y_12_ antagonist treatment on α‐granule secretion and the formation of integrin α_IIb_β_3_ in rabbit platelets were measured by flow cytometry. α‐granule secretion was determined by measurement of P‐selectin (CD62P) expression on the platelet surface. Integrin α_IIb_β_3_ expression was measured by the relative binding of fibrinogen‐FITC. Treatment with DT‐678, clopidogrel, and ticagrelor dose‐dependently decreased both α‐granule secretion and the formation of integrin α_IIb_β_3_ on platelets in response to ADP activation compared to vehicle (Figure 2). Ex vivo activation of platelets from vehicle‐treated animals resulted in 33.36 ± 5.49% double‐positive cells (CD62^+^fibrinogen^+^), while double‐positive platelets from animals with the highest doses of antagonists were significantly lower (5.96 ± 1.31%, 7.38 ± 1.88%, and 9.82 ± 1.41% for DT‐678, clopidogrel, and ticagrelor, respectively).

### 3.2 Ex vivo platelet aggregation is dose‐dependently inhibited by P2Y_12_ antagonist treatment

**Figure 3:**
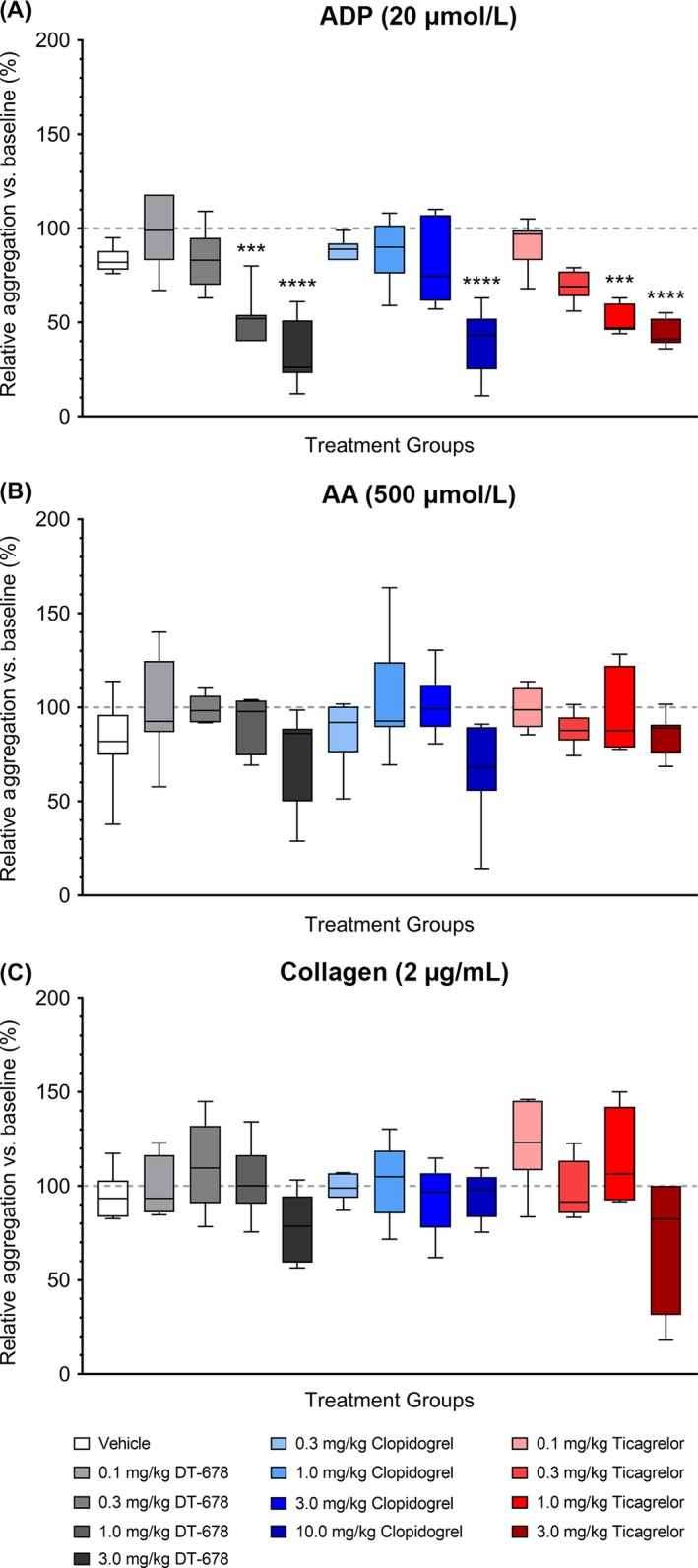
Percent platelet aggregation responses to (A) ADP (20 µmol/L), (B) AA (500 µmol/L), and (C) collagen (2 µg/mL) for animals treated with vehicle, DT‐678 (0.1, 0.3, 1.0, or 3.0 mg/kg), clopidogrel (0.3, 1.0, 3.0, 10.0 mg/kg), or ticagrelor (0.1, 0.3, 1.0, or 3.0 mg/kg). Blood was collected before and 2 h after the administration of drugs. The data are presented as a box and whisker plot of percent change relative to baseline and represent data from five to seven separate experiments. The middle line indicates the median and the lower and upper bars represent the minimum and maximum values, respectively. The box extends from the 25th to the 75th percentiles. ***P < .001, ****P < .0001 when compared with vehicle‐treated group by one‐way ANOVA followed by Dunnett's post hoc test. ADP, adenosine diphosphate; AA, arachidonic acid

Ex vivo aggregation of platelets was measured by light transmission aggregometry using platelets isolated from blood drawn from rabbits treated with different concentrations of DT‐678, clopidogrel, or ticagrelor. ADP (20 µmol/L)‐induced platelet aggregation was dose‐dependently inhibited by treatment with DT‐678 (27.2 ± 6.4%), clopidogrel (34.4 ± 5.9%), and ticagrelor (41.6 ± 2.7%) compared to vehicle (83.6 ± 3.5%; Figure 3A). However, AA‐ (500 µmol/L) and collagen (2 µg/mL)‐induced aggregations were relatively unaffected (Figure 3B,C, respectively). While maximum inhibition of ADP‐induced aggregation was observed 2 hours after the administration of drugs, similar results were recorded at 10 minutes and 1 hour (Figure S1). No change in AA‐ or collagen‐induced aggregation was detected at any time point (Figures S2 and S3, respectively).

### 3.3 Tongue bleeding time is significantly prolonged by treatment with clopidogrel and ticagrelor, but not DT‐678

**Figure 4:**
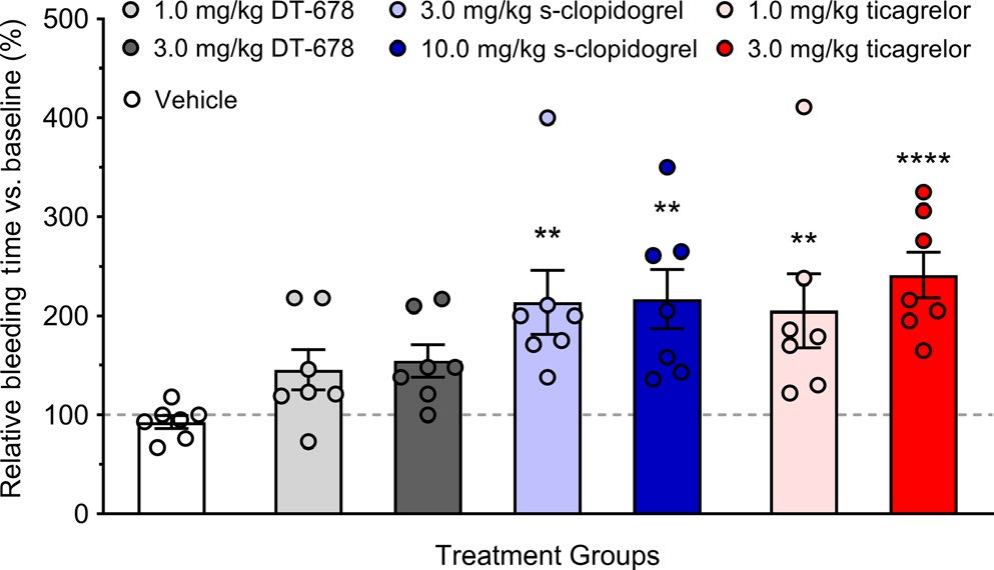
Tongue bleeding time after treatment with vehicle, DT‐678 (1.0 or 3.0 mg/kg), clopidogrel (3.0 or 10.0 mg/kg), or ticagrelor (1.0 or 3.0 mg/kg). Bleeding times were assessed at baseline and 2 h after the administration of drugs. The data are presented as percent change relative to baseline and represent the mean of seven experiments ± SEM. **P < .01, ****P < .0001 when compared with vehicle‐treated group by one‐way ANOVA followed by Dunnett's post hoc test

Tongue template bleeding time was assessed using a Surgicutt^®^ device. Bleeding times were similar at baseline in all the treatment groups (data not shown). Treatment with antiplatelet doses of clopidogrel (3.0 and 10.0 mg/kg; 231.4 ± 42.8% and 235.4 ± 38.2%, respectively) and ticagrelor (1.0 and 3.0 mg/kg; 216.0 ± 53.0% and 265.6 ± 23.9%, respectively) significantly prolonged bleeding times 2 hours after treatment compared to vehicle (92.2 ± 9.2%; Figure 4). Treatment with antiplatelet doses DT‐678 (1.0 and 3.0 mg/kg; 155.6 ± 28.1% and 172.2 ± 17.0%, respectively) modestly prolonged bleeding time, but the difference was not statistically significant. Subplatelet inhibitory doses of all the drugs did not increase the bleeding time (Figure S4).

## 4 DISCUSSION

Antagonists of purinergic P2Y_12_ receptors are an important component in the pharmacological management of patients at risk for thrombotic events. Prescribed together with low‐dose aspirin, these agents have been proven effective at reducing the risk of heart attack and stroke. Despite the availability of multiple P2Y_12_ antagonists recommended for DAPT, interpatient variability persists in a significant number of ACS patients, which leads to increased risk of ischemic complications and reduced survival rate. Although clopidogrel is generally effective and well tolerated, it has well‐documented clinical limitations such as interpatient variability, delayed onset of action, and drug‐drug interactions. Approximately, 30% of Caucasians and up to 70% of Asians are resistant to clopidogrel.^14^, ^19^, ^20^ Genetic polymorphisms in CYP2C19 are a main contributor to this lack of responsiveness. Patients who carry CYP2C19 loss‐of‐function mutations fail to effectively metabolize the clopidogrel prodrug to the pharmacologically AM. More recently a reversible P2Y_12_ antagonist, ticagrelor, has been developed. This agent differs from previous thienopyridine agents in that it is not a prodrug and therefore does not require bioactivation. Ticagrelor, however, is subject to CYP3A4‐mediated metabolism and its primary metabolite is also a potent P2Y_12_ inhibitor.^21^ Ticagrelor is prone to numerous adverse drug interactions due to the induction and inhibition of CYP3A4 by many clinically used drugs including ketoconazole, atazanavir, ritonavir, rifampin, dexamethasone, statins, and more.^22^ Although the newer agents like ticagrelor have improved clinical outcomes, they also increase the risks of bleeding.^23^, ^24^ The primary safety concern with ticagrelor is bleeding as indicated in the PLATO trial supporting the approval of ticagrelor by the FDA.^23^ Patients taking ticagrelor are nine times more likely to discontinue the use of drug than those on clopidogrel.

Due to the numerous limitations with P2Y_12_ antagonists, our research team has developed a novel conjugate of clopidogrel that spontaneously and nonenzymatically releases the AM after oral and intravenous administration.^16^, ^17^, ^18^ Our earlier studies have demonstrated the rapid release of the AM within minutes of administration.^18^ In this study, we report our findings comparing the antiplatelet and bleeding effects of DT‐678 to clopidogrel and ticagrelor. The inhibitory effects of these agents on platelet activation were evident in the reduced surface expression of P‐selectin and decreased binding of fluorescently labeled fibrinogen in response to ADP activation. In addition, ADP‐induced platelet aggregation was dose‐dependently inhibited by treatment with DT‐678, clopidogrel, and ticagrelor. Importantly, however, tongue template bleeding times were only significantly prolonged by treatment of clopidogrel and ticagrelor and not DT‐678 suggesting that the latter has a more favorable safety profile.

Preclinical assessment of bleeding risk is limited by the availability of standardized animal models. A great deal of effort has been devoted to characterizing the murine tail cut assay in assessing the bleeding tendency of antithrombotic drugs and genetic hemostatic disorders. The severity of the tail amputation, however, does not accurately replicate the clinical state. Furthermore, there is no common protocol for testing and as a result bleeding times vary considerably between laboratories. This inconsistency makes direct comparison of the adverse bleeding effects of drugs difficult.^25^, ^26^ Template bleeding tests in humans were first described by Milian in 1901 and were later improved by several others.^27^, ^28^, ^29^ The tests involve making a small incision on the skin (often the forearm) and recording the time required for blood flow to cease. Due to the global coverage of fur on the body of most laboratory animal species, our laboratory has adapted this assay to use the upper surface of the tongue while the animal is under anesthesia.^30^, ^31^ We have routinely used bleeding devices that create a reproducible incision with respect to length and depth allowing for reliable comparisons between time points and animals possible. While this model does not accurately mimic spontaneous bleeding in humans, the severity of the incision more closely reflects clinical observations.

In the current study, tongue bleeding times were significantly increased in animals treated with FDA‐approved P2Y_12_ antagonists, but not DT‐678 which possesses the same pharmacological cargo as clopidogrel. The doses of agents used in this study were based on their antiplatelet efficacy which was empirically determined by flow cytometry and platelet aggregation. These results are in partial agreement with our previously reported bleeding data.^18^ In that study, no increase in bleeding time was observed with DT‐678 at a dose of 1 mg/kg; however, there was a significant increase in bleeding at a dose of 2 mg/kg. At present, we do not have an explanation for the observed differences in bleeding. However, no comparative analysis was performed with other P2Y_12_ antagonists in the previous study and therefore it is unknown whether clopidogrel or ticagrelor would have further prolonged the bleeding time.

There are multiple explanations for the observed bleeding results in the present study. Clopidogrel undergoes a complicated metabolism pathway in which at least 15 different compounds are created in addition to the canonical AM. Literature evidence suggests that some of these compounds may possess biological activity. In fact, a metabolite has recently been identified by Zhu and colleagues^32^ which possesses thiol‐mediated antiplatelet activity separate from the inhibition of P2Y_12_. The additive effects of these “nonactive” metabolites of clopidogrel may therefore potentiate bleeding in animals and humans.

With respect to ticagrelor, the compound has been reported to have potential off‐target effects on purinergic receptors in the vasculature leading to vasodilation.^33^, ^34^ Ticagrelor is structurally distinct from the thienopyridine class of antiplatelet agents and as such may possess differential actions at purinergic receptors in distinct tissues. In addition, increased circulating adenosine concentrations have also been reported in patients taking ticagrelor which might also explain some of the vascular effects associated with the drug.^35^ It is possible that these vascular properties of ticagrelor may result in an increased bleeding tendency in the presence of simultaneous inhibition of platelet P2Y_12_. The importance of these effects is uncertain, however, as more recent reports in humans suggest no difference in the vascular effects of thienopyridines and ticagrelor.^36^

An interesting observation from our results is that clopidogrel required an approximately 10‐fold higher dosage than DT‐678 to elicit similar antiplatelet effects. A likely explanation for this finding is that the clopidogrel prodrug undergoes a complicated metabolism pathway in which only 1%‐5% of administered dose are converted to the AM.^37^, ^38^ DT‐678, on the other hand is nonenzymatically converted to the AM and therefore all the administered dose is available for inhibition of P2Y_12_. This finding is potentially important in the context of Type II diabetic patients treated with DAPT. This subset of patients has an impaired ability to form the AM of thienopyridine P2Y_12_ antagonists.^39^, ^40^ The underlying effect is hypothesized to result from dysregulation of cytochrome P450 (CYP450) enzymes.^41^, ^42^ CYP450‐independent activation of the AM as with DT‐678 may find utility in this unique population. It is important to note, however, that in the present study, rabbits were treated with a single intravenous injection of each drug. Further investigation is required to determine whether the observed effects persist with chronic oral administration.

We conclude that in an experimental bleeding model in rabbits, DT‐678 did not significantly prolong bleeding time at doses that were capable of inhibiting platelet activation and aggregation. Conversely, administration of clopidogrel or ticagrelor significantly prolonged bleeding time at equally effective antiplatelet doses. Given its simplified activation pathway and favorable pharmacokinetics, our results suggest that DT‐678 is a potentially useful alternative to existing P2Y_12_ antagonists with improved predictability and safety.

## AUTHOR CONTRIBUTIONS

*Participated* *in* *research* *design*: D. A. Lauver, BE Markham, Y. E. Chen, H. Zhang. *Conducted* *experiments*: D. A. Lauver, D. S. Kuszynski, B. Christian, M. P. Bernard, J. P. Teuber. *Performed* *data* *analysis*: D. A. Lauver, D. S. Kuszynski, B. Christian, M. P. Bernard. *Wrote* *or* *contributed* *to* *the* *writing* *of* *the* *manuscript*: D. A. Lauver, D. S. Kuszynski, M. P. Bernard, B. E. Markham, Y. E. Chen, H. Zhang.
