# Supplementary figures and images for: DT‐678 inhibits platelet activation with lower tendency for bleeding compared to existing P2Y12 antagonists
Source: Pharmacol Res Perspect. 2019 Jul 25;7(4):e00509. doi: 10.1002/prp2.509 (PMC6658415; doi:10.1002/prp2.509)

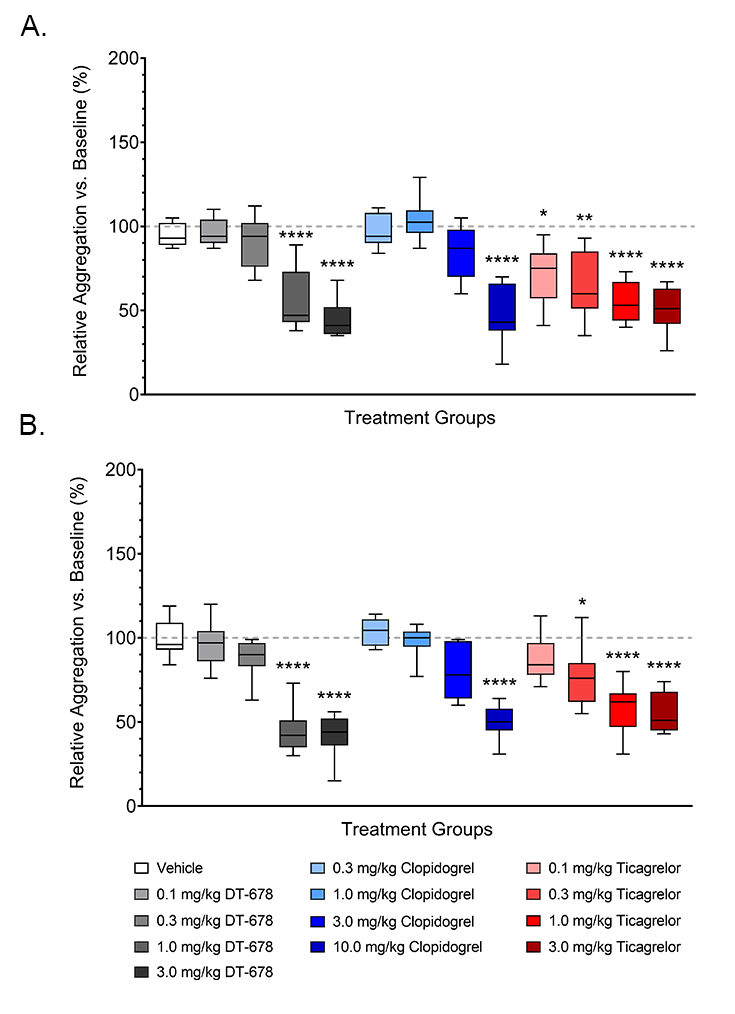

Supplement: Supplementary file 1 [file PRP2-7-e00509-s001.tif]

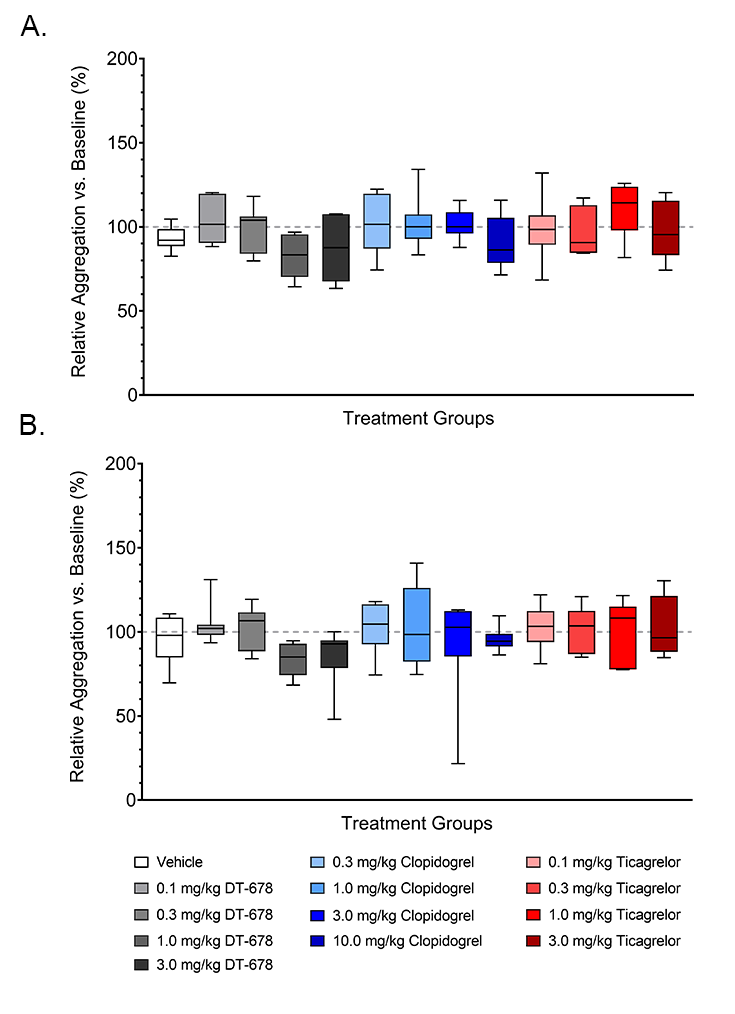

Supplement: Supplementary file 2 [file PRP2-7-e00509-s002.tif]

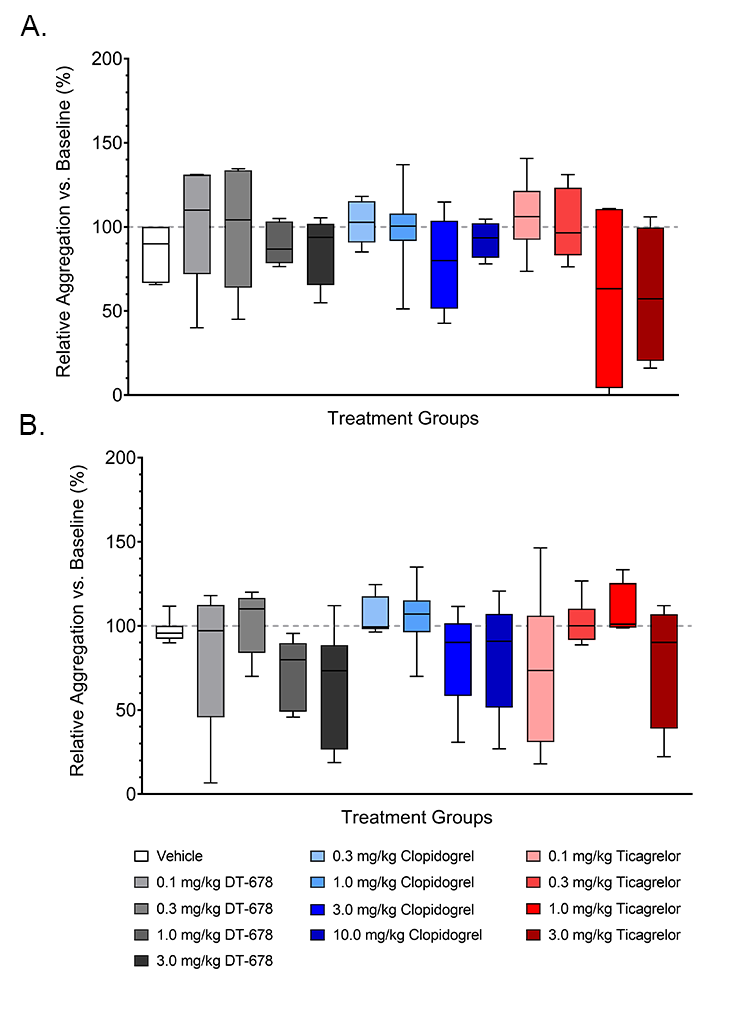

Supplement: Supplementary file 3 [file PRP2-7-e00509-s003.tif]

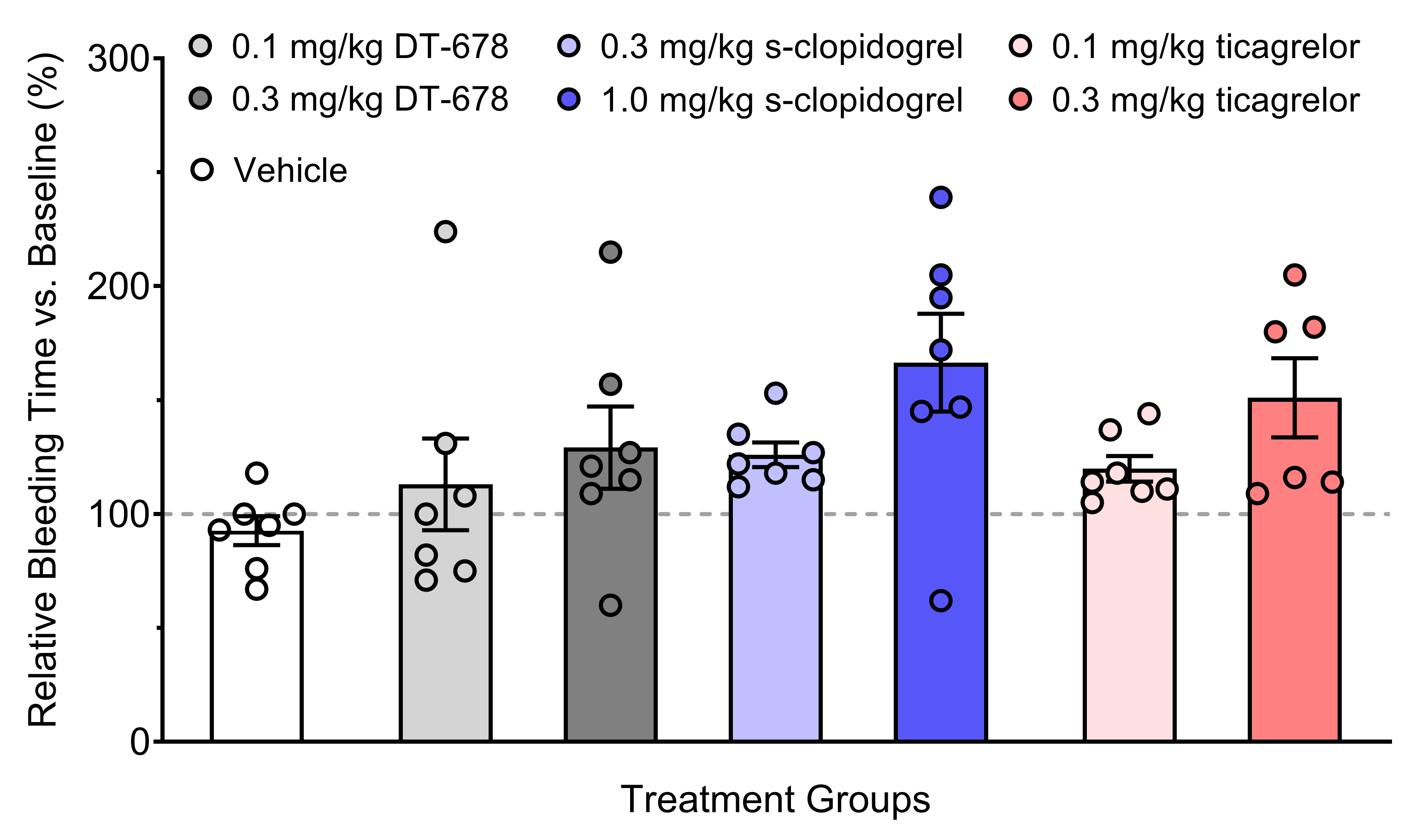

Supplement: Supplementary file 4 [file PRP2-7-e00509-s004.tif]
