# Supplementary material for: DT‐678 inhibits platelet activation with lower tendency for bleeding compared to existing P2Y12 antagonists
Source: Pharmacol Res Perspect. 2019 Jul 25;7(4):e00509. doi: 10.1002/prp2.509 (PMC6658415; doi:10.1002/prp2.509)
Supplement: Supplementary file 5 [file PRP2-7-e00509-s005.docx]

**Supplemental Figure Captions**

Supplemental Figure 1. Percent platelet aggregation responses to adenosine diphosphate (20 µM) for animals treated with vehicle, DT-678 (0.1, 0.3, 1.0 or 3.0 mg/kg), clopidogrel (0.3, 1.0, 3.0, 10.0 mg/kg) or ticagrelor (0.1, 0.3, 1.0 or 3.0 mg/kg). Blood was collected before, and 10 minutes or 1 hour after that administration of drugs. The data are presented as a box and whisker plot of percent change relative to baseline (before treatment) for (A) 10 minutes and (B) 1 hour after the administration of drugs. The values represent data from 7 separate experiments. The middle line indicates the median and the lower and upper bars represent the minimum and maximum values, respectively. The box extends from the 25th to the 75th percentiles. * p < 0.05, ** p < 0.01, **** p <0.0001 when compared with vehicle treated group by one-way ANOVA followed by Dunnett’s post hoc test.

Supplemental Figure 2. Percent platelet aggregation responses to arachidonic acid (500 µM) for animals treated with vehicle, DT-678 (0.1, 0.3, 1.0 or 3.0 mg/kg), clopidogrel (0.3, 1.0, 3.0, 10.0 mg/kg) or ticagrelor (0.1, 0.3, 1.0 or 3.0 mg/kg). Blood was collected before, and 10 minutes or 1 hour after that administration of drugs. The data are presented as a box and whisker plot of percent change relative to baseline (before treatment) for (A) 10 minutes and (B) 1 hour after the administration of drugs. The values represent data from 5-7 separate experiments. The middle line indicates the median and the lower and upper bars represent the minimum and maximum values, respectively. The box extends from the 25th to the 75th percentiles.

Supplemental Figure 3. Percent platelet aggregation responses to collagen (2.0 µg/ml) for animals treated with vehicle, DT-678 (0.1, 0.3, 1.0 or 3.0 mg/kg), clopidogrel (0.3, 1.0, 3.0, 10.0 mg/kg) or ticagrelor (0.1, 0.3, 1.0 or 3.0 mg/kg). Blood was collected before, and 10 minutes or 1 hour after that administration of drugs. The data are presented as a box and whisker plot of percent change relative to baseline (before treatment) for (A) 10 minutes and (B) 1 hour after the administration of drugs. The values represent data from 5-7 separate experiments. The middle line indicates the median and the lower and upper bars represent the minimum and maximum values, respectively. The box extends from the 25th to the 75th percentiles.

Supplemental Figure 4. Tongue bleeding time after treatment with vehicle, DT-678 (0.1 or 0.3 mg/kg), clopidogrel (0.3, 1.0 mg/kg) or ticagrelor (0.1 or 0.3 mg/kg). Bleeding times were assessed at baseline and 2 hours after the administration of drugs. The data are presented as percent change relative to baseline and represent the mean of 7 experiments ± SEM.
